# Predicting Fluid Responsiveness Using Bedside Ultrasound Measurements of the Inferior Vena Cava and Physician Gestalt in the Emergency Department of an Urban Public Hospital in Sub-Saharan Africa

**DOI:** 10.1371/journal.pone.0162772

**Published:** 2016-09-27

**Authors:** Hendry Robert Sawe, Cathryn Haeffele, Juma A. Mfinanga, Victor G. Mwafongo, Teri A. Reynolds

**Affiliations:** 1 Emergency Medicine Department, Muhimbili University of Health and Allied Sciences, Dar es salaam, Tanzania; 2 Emergency Medicine Department, Muhimbili National Hospital, Dar es Salaam, Tanzania; 3 School of Medicine, University of California Los Angeles, Los Angeles, California, United States of America; 4 Department of Emergency Medicine, University of California San Francisco, San Francisco, California, United States of America; The University of Tokyo, JAPAN

## Abstract

**Background:**

Bedside inferior vena cava (IVC) ultrasound has been proposed as a non-invasive measure of volume status. We compared ultrasound measurements of the caval index (CI) and physician gestalt to predict blood pressure response in patients requiring intravenous fluid resuscitation.

**Methods:**

This was a prospective study of adult emergency department patients requiring fluid resuscitation. A structured data sheet was used to record serial vital signs and the treating clinician’s impression of patient volume status and cause of hypotension. Bedside ultrasound CI measurements were performed at baseline and after each 500mL of fluid. Receiver operating characteristic (ROC) curve analysis was performed to characterize the relationship between CI and Physician gestalt, and the change in mean arterial pressure (MAP).

**Results:**

We enrolled 364 patients, 52% male, mean age 36 years. Indications for fluid resuscitation were haemorrhage (54%), dehydration (30%), and sepsis (17%). Receiver operating characteristic curve analysis found optimal CI cut-off values of 45%, 52% and 53% to predict a MAP rise of 5, 8 and 10 mmHg per litre of fluid, respectively. The sensitivity and specificity of CI of 50% for predicting a 10mmHg increase in MAP per litre were 88% (95%CI 81–93%) and 73% (95%CI 67–79%), respectively, area under the curve (AUC) = 0.85 (0.81–0.89). The sensitivity and specificity of physician gestalt estimate of volume depletion severity were 68% (95%CI 60–75%) and 86% (95%CI 80–90%), respectively, AUC = 0.83 (95% CI: 0.79–0.87). Those with a baseline CI ≥ 50% (51% of patients) had a 2.8-fold greater fluid responsiveness than those with a baseline CI<50% (p<0.0001).

**Conclusion:**

Ultrasound measurement of the CI can predict blood pressure response among patients requiring intravenous fluid resuscitation and may be useful in early identification of patients who will benefit most from volume resuscitation, and those who will likely require other interventions.

## Introduction

Hypotension in the emergency department has been shown to be an independent predictor of in-hospital mortality and, if untreated, can progress to shock, a medical emergency, which carries a substantial mortality [1–5]. Early recognition and early intervention are vital to prevent progression of hypotension to shock and complete cardiovascular collapse. Intravenous fluids administration remains the cornerstone of the management of patients with hypovolemia and/or shock [6–8]. Several studies and international consensus guidelines suggest that accurate prediction of patient response to fluid challenge is a crucial element in optimizing clinical outcome with fluid resuscitation [9–11]. Both under and over resuscitation with fluid may cause poor clinical outcome [12,13]. Under-resuscitation may cause tissue hypoperfusion and result in worsening organ dysfunction, while over-resuscitation may lead to pulmonary oedema resulting in poor oxygen delivery to tissues and organs. Fluid administration and dosing during resuscitation of hypovolemic patients has largely been based on clinical examination or, in high resource centres, by monitoring of central venous pressure (CVP) [10,14]. However, clinical examination may not provide a reliable estimate of volume status, and monitoring of central venous pressure (CVP) is expensive, invasive, requires highly trained staff and has itself been shown to be unreliable as a surrogate for volume status [15–17].

The inferior vena cava (IVC) is a very compliant vessel whose size varies with changes in intravascular pressure making it possible for sonographic evaluation of the IVC to provide a non-invasive measure of volume status [18,19]. The IVC collapsibility or Caval Index (CI) is calculated as relative change in IVC diameter during one respiratory cycle, and has been shown to correlate with CVP [20–22]. A CI of greater than or equal to 50% has been shown to correlate strongly with a CVP of less 8mmHg, a cutoff commonly used to determine the need for volume resuscitation [23].

Various studies on the use of bedside ultrasound in the Emergency Department have shown that ultrasound assessment of IVC dimensions can be performed by operators with limited echocardiographic experience in a busy outpatient department using handheld ultrasound devices [24,25]. In areas where resources are limited and transfer may be required for definitive care, bedside ultrasound can have an even greater impact by facilitating critical early diagnosis and initial resuscitation [26]. In many areas of sub-Saharan Africa, bedside ultrasound may be the only economically viable and sustainable modality of imaging [27–29].

The primary aim of this investigation was to compare the mean arterial blood pressure response to fluid rescucitation in patients with a baseline caval indices greater than or equal to 50% and to those with Caval Index of less 50%. Secondary aims include determining the relationship between bedside ultrasound measurement of CI and the physician’s impression of the patient’s baseline volume status.

## Materials and Methods

### Study design

This was a prospective observational study of a convenience sample of adult patients presenting to the Emergency Medicine Department (EMD) of Muhimbili National Hospital (MNH) from May 2012 to October 2012.

### Study setting and Population

The study was conducted in the EMD-MNH in Dar es Salaam, Tanzania. The EMD was established in 2010 as the first, and is still the only, full capacity public emergency department in Tanzania. MNH is the national referral care centre with a bed capacity of 1500 and serves as the top referral hospital for all hospitals in Tanzania. At the time of this study, board-certified visiting emergency physicians from the USA, Canada and South Africa provided clinical supervision and teaching to intern doctors (recent medical school graduates), registrars (general practitioners who have completed internship) and emergency medicine residents (in a 3-year residency program). The department has annual volume of around 36,000 patients.

In this study all adult patients clinically judged by their treating physician to require intravenous fluid resuscitation, were eligible for enrollment. We excluded patients if the volume resuscitation was begun prior to notification of research personnel.

### Study protocol

Screening and enrollment was completed by one investigator (HRS), who completed a structured data sheet, recording basic patient demographic information, serial measurements of the IVC diameter, vital signs and the treating physician’s gestalt estimate of the patient’s volume depletion. The clinicians were asked to provide their clinical gestalt impression of patient’s volume depletion as mild, moderate or severe based on the history and physical examination. This clinical impression was sought and recorded just prior to initiation of intravenous fluid (IVF) administration to patients. The same clinician was then asked what he/she believed to be the main cause of volume depletion, and which elements of the physical examination influenced the decision to give fluid to the patient. Patients were enrolled consecutively at times when the investigator (HRS) was available for data collection shifts, and included day night, weekday and weekend hours.

### Measurements

All IVC measurements were performed by using the M-Turbo ultrasound (SonoSite, Inc. Bothell, Washington USA). The ultrasound was available in the EMD and was being used as part of usual clinical care. The researcher received specific didactic and practical training in the use of bedside ultrasound for IVC measurement. He had succesfully completed the training program requirement for basic emergency ultrasound and had been working with bedside ultrasound in his clinical duties for more than two years prior to initiation of the study. All study measurements (still images, video clips and m-mode) were recorded and reviewed by a Board-certified and ultrasound fellowship-trained Emergency Medicine physician for accuracy of image acqusition and measurement technique.

With patients lying in supine position, the maximum and minimum IVC diameter (across the respiratory cycle), were measured at 2 to 3 cm from the right atrial border in a long-axis/subxiphoid view. All patients underwent ultrasound measurements before the initiation of IVF infusion, and after each 500ml of fluid administered for the duration of the initial infusion; measurements were taken after each 500ml up to, but not beyond, 2000ml.

The volume infused was determined by the treating clinician. The researcher recorded serial vital sign measurements concurrent with each ultrasound measurement.

### Data analysis

Data from handwritten data sheets was transferred into an Excel spreadsheet (Microsoft Corporation, Redmond, WA, USA) and then imported into SAS Software, version 9.3 (SAS Institute Inc, Cary, NC, USA). Procedure, frequency and univariate functions were performed to check for any outliers and clean the dataset. Patient descriptive characteristics, mean arterial pressure (MAP) and CI are reported, including means, medians, and standard deviations. Receiver operating characteristic (ROC) curve analysis was performed to characterize the relationship between CI and Physician gestalt estimate of severity of dehydration, and the change in MAP of 5, 8 or 10 mmHg per litre of fluid. Furthermore, a subgroup analysis by indication for resuscitation (sepsis, dehydration and haemorrhage) was performed to predict a raise in MAP of 10mmHg per litre of fluid, at a CI cut-off value of 50%. The CI was categorized into a bivariate (greater than 50% and less or equal to 50%) variable and used when stratifying each patient's change in MAP per unit volume of fluid infused. T-tests were used to compare fluid responsiveness between groups. Patients were also stratified based on the physician clinical assessment of volume depletion.

### Ethics Approval

The study protocol was reviewed and approved by the Institutional Review Board of the Muhimbili University of Health and Allied Sciences (MUHAS) and the committee on human research of the University of California, San Francisco. Informed written consent was provided by patients or their proxies.

## Results

### Study population demographics and average baseline values

We enrolled 364 patients from May 12^th^ to October 2^nd^, 2012 with a male to female ration of approximately one, and more than half of the patients enrolled were below 40 years of age. The mean CI at presentation was less than 50% and on average patents received about one litre of intravenous crystalloids bolus. (Table 1).

**Table 1:** Study population demographics and baseline variables.

**Patient Demographics**	**N = 364**
**Age (years)**	
Mean (±SD)	36 ± 12
**Gender**	
Male	190 (52.2%)
Female	174 (48.8%)
**Patients baseline variables**	**Mean (SD)**
Mean Arterial Pressure (MAP)	73 (11 mmHg)
Caval Index (CI)	47.7% (19.3%)
IV fluid bolus given to patients	1050 (443 ml)

### Indications for volume resuscitation

The most frequent indication for volume resuscitation of patients as stated by the clinicians was haemorrhage, while sepsis was the least. The dehydration category included diarrhoea 18/364 (4.9%), poor oral intake 39/364 (10.7%) and vomiting 51/364 (14.0%). Hypotension was the most frequent physical examination finding reported by the clinicians and decreased skin turgor was the least frequent (Table 2).

**Table 2:** Indications for volume resuscitation.

**Clinicians' stated causes of volume depletion**	**N = 364**
Haemorrhage	195 (53.6%)
Dehydration*	108 (29.7%)
Sepsis	61 (16.8%)
**Physical examination suggestive of hypovolemia**	**N = 364**
Hypotension	151 (41.5%)
Tachycardia	110 (30.2%)
Dry mucous membrane	90 (24.7%)
Decreased skin turgor	13 (3.6%)

* Includes: Diarrhoea 18/364 (4.9%), Poor oral intake 39/364 (10.7%) and vomiting 51/364 (14.0%).

### Clinician estimation of patient volume status

Clinicians estimated 40.1% of patients to be mildly dehydrated, and 35.7% of patients to be severely dehydrated (Table 3). Overall, the CI was lower and volume responsiveness higher in patients who clinicians rated as moderately and severely dehydrated as compared with those rated mild.

**Table 3:** Clinician estimation of patient volume status and baseline CI.

		Baseline Caval Index		Change in MAP per unit volume of IVF	
Physician assessment	Percentage (N = 364)	Median (%)	Range (%)	Median (mmHg/L)	Range (mmHg/L)
Mild	146 (40.1%)	30	9–80	4	(-24)–33
Moderate	88 (24.2%)	53	6–83	6	0–29
Severe	130 (35.7%)	62	22–95	12	(-10)–28

### The relationship between caval index measurements and fluid responsiveness

Subjects were initially divided into two groups, those with CI less than fifty percent (CI< 50%) and those with CI greater or equal to fifty percent (CI ≥ 50%), giving a ratio of 0.95. The patients with a CI ≥ 50% had a 2.8 fold (p<0.0001) greater fluid responsiveness than patients with a CI <50% (Table 4).

**Table 4:** Relationship between Caval Index and fluid responsiveness.

Baseline Caval Index		Change in MAP per unit volume (mmHg/L)	Confidence Interval	
	N = 364	Mean (±SD)	95% CI	p-value^α^
CI<50%	177 (48.6%)	4.0 (± 5.6)	3.6–5.1	
				<0.0001
CI≥50%	187 (51.4%)	11.3 (± 6.3)	10.8–12.1	

^α^Statistical significance: p<0.05

### Change in MAP per unit volume of fluid

Within each group (those with CI ≥ 50% and those with CI<50%), patients who received only 500ml of IVF had a similar increase in MAP per unit volume as patients who received more than 500 ml (Table 5).

**Table 5:** Change in MAP per unit volume of fluid.

Baseline Caval Index		Change in MAP after 500ml	Change in MAP after 1000ml	Change in MAP after 1500ml	Change in MAP after 2000ml
	N = 364	Mean (±SD)	Mean (±SD)	Mean (±SD)	Mean (±SD)
CI<50%	N = 177	4.0 (±6.9)	6.2 (±4.2)	5.2 (±4.0)	5.5 (±2.2)
CI≥50%	N = 187	10.7 (±7.8)	13.1 (±7.0)	10.0 (±4.8)	10.0 (±3.2)

### The relationship between CI, Physician gestalt and change in MAP per unit volume

To further characterise the relationship between CI and MAP, we performed receiver operating characteristic (ROC) curve analysis to investigate the ability of baseline CI to predict a rise in MAP of 5, 8 or 10 mmHg per litre of fluid. At these cut-offs, we found the area under the curve (AUC) to be 0.83, 0.84 and 0.84 and the optimal CI cut-off value to be 45%, 52% and 53%, respectively. Using our pre-specified CI cut-off of 50%, the test characteristics for predicting a 10mmHg increase in MAP per litre were a sensitivity of 87.7% (95%CI 81.2–92.5%), specificity of 72.9% (95%CI 66.5–78.7%), positive likelihood ratio of 3.2 (95%CI 2.6–4.1) and negative likelihood ratio 0.2 (95%CI 0.1–0.3).

**Fig 1:**
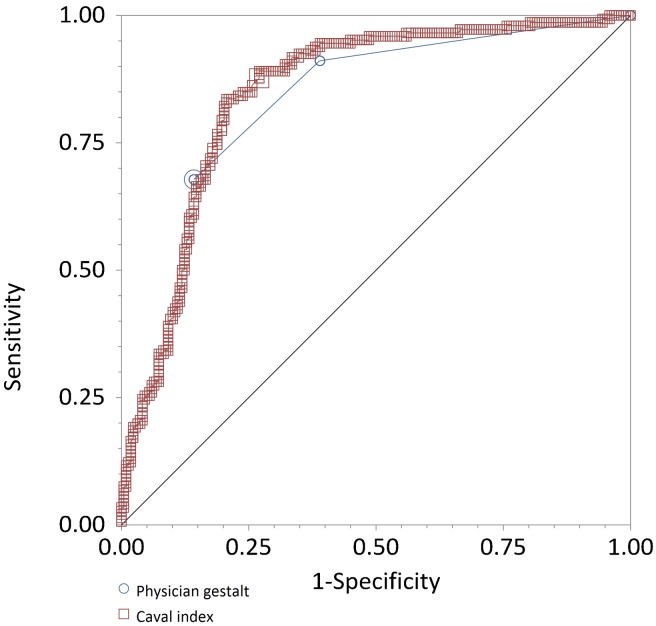
Relationship between CI, Physician gestalt and change in MAP per unit volume.

The ROC curve analysis of physician gestalt estimate of severity of dehydration (mild, moderate or severe) to predict a raise in MAP of 10mmHg per litre of fluid, found the AUC of 0.83 (95% CI 0.79–0.87%). The corresponding test characteristics were sensitivity of 67.8% (95%CI 59.6–75.3%), specificity of 85.8% (95%CI 80.4–90.1%), positive likelihood ratio of 4.8 (95%CI 3.4–6.8) and negative likelihood ratio of 0.4 (95%CI 0.3–0.5) (Fig 1).

Subgroup analysis by indication for resuscitation revealed AUCs of 0.89 (95%CI 0.82–0.97), 0.86 (95%CI 0.78–0.95), 0.80 (95%CI 0.73–0.86), to predict a raise in MAP of 10mmHg per litre of fluid, at a CI cut-off value of 50% for sepsis, gastrointestinal symptoms and haemorrhage respectively.

## Discussion

During resuscitation of critically ill patients who require IVF, it is important to predict which patients will have an increase in MAP in response to volume expansion and which may be at risk of harmful volume overload. Prediction of fluid responsiveness is crucial, as this will guide intervention aimed at increasing the overall cardiac output, tissue perfusion and oxygen delivery [6,8,30].

Overall, haemorrhage was the indication for volume resuscitation in over half of the study patients. The majority of patients who received fluid had a physical examination finding of hypotension and the baseline MAP of the study population was 73 mmHg. A range of clinical signs and symptoms have been proposed in the literature as appropriate indicators and predictors of volume depleted status [31,32]. However, similar to previous literature, our results (see the ROC curve analysis of physician gestalt estimate of severity of dehydration) suggest that clinical examination may not provide a reliable enough estimate of volume status [33,34]. The physician estimation of volume status revealed that despite a trend towards an increase in the CI in the patients rated as moderately to severely volume depleted by clinicians, there was substantial overlap in the groups, limiting the utility of clinical estimation to predict volume responsiveness. Similar to previously published studies [23], we have reported CI as a binary measurement in relation to change in MAP per unit volume for each of the CI categories.

The findings of our study suggests that the majority of patients who are judged by clinicians to be hypovolemic and hence requiring fluid resuscitation did not come close to reaching the target of the recommended initial bolus of intravenous crystalloids in hypotensive patients. These patients were substantially under-resuscitated, with only 4 patients in the CI < 50% category receiving the recommended initial bolus of 2000 ml. As more than half of all patients only received between 500ml to 1000ml as an initial bolus, this may have affected the effect observed change in MAP within each group. These findings are similar to an observational study by de Valk et al in the Netherlands in a group of patients with shock receiving serial IVC measurements: poor blood pressure response in those with a high CI was attributed to low fluid bolus [35].

Overall, the mean volume of the initial intravenous fluid resuscitation was progressively higher in patients categorized by treating physician as being severely volume depleted as compared to those categorized as moderate or mild.

Our analysis shows that a CI of greater than or equal to fifty percent (CI ≥ 50%) is indeed predictive of greater fluid responsiveness to initial bolus of 500ml to 2000ml than a CI of less than fifty percent (CI<50%), with patients in the CI ≥50% category having a change in MAP of 11 mmHg per litre of fluid compared to those in the CI<50% category who had a MAP change of 4 mmHg per litre of fluid. Previous literature suggest that, a change in MAP of greater or equal to 10 mmHg is considered clinically significant, whereas a change of 5 mmHg is considered not clinically significant [36].

These results study suggest that bedside ultrasound may be a very useful tool for rapidly stratifying those patients whose hypotension is likely be responsive to initial fluid bolus as early identification, stratification and intervention is essential to improve outcomes [6].

Subgroup analysis revealed that, patients who were identified to have sepsis as the indication for resuscitation had a better response to fluid challenge compared to those with gastrointestinal symptoms and haemorrhage.

Our ROC curve analysis of relationship between CI and MAP, suggests an optimal cut of value similar to our hypothesized value of 50%, further confirming the hypothesized binary value which has been proposed in previous studies [23,37]. Different studies published in the critical care literature have described a range of implications of CI measurements [35,38–40]. To our knowledge, this is the first physiological study to report on the relationship between serial measurements of CI and dynamic changes in MAP per unit volume in patients requiring fluid resuscitation in low resource settings. Our findings provide an opportunity for studies to establish the feasibility of the use of bedside ultrasound in clinical practice by all providers in resource-limited settings.

## Limitations

Given study resource constraints, we were limited to convenience sampling in a single tertiary centre, and this limits the generalizability of our results. To limit potential bias, we did use consecutive enrolment of all patients available during the data collection periods.

The ultrasound measurements were performed by a single emergency physician, and so we were unable to measure inter-rater reliability; This might limit generalizability to other providers. However, prior work has demonstrated good inter-observer agreement for visual estimation of CI, with a weighed Cohen’s kappa of 0.78 (95%CI (0.67–0.89) [41]. Quantitative measurements should only be more accurate. In addition, to mitigate the effect of a single observer, each measurement was performed by on screen caliper and recorded, and then independently verified by a fellowship-trained emergency ultrasound specialist for positioning and technique.

As this was an observational study, the volume of fluid resuscitation was determined by the treating physicians’ clinical judgment, limiting our analysis to the actual volume infused per patient, which ranged from 500ml to 2000ml.

## Conclusions

Ultrasound measurement of the CI can predict blood pressure response among patients requiring intravenous fluid resuscitation and may be useful in early identification of patients who will benefit most from volume resuscitation, and those who will likely require other interventions.

## Supporting Information

S1 File IVC Data-HRS. (PDF)
