# Supplementary material for: Predicting Fluid Responsiveness Using Bedside Ultrasound Measurements of the Inferior Vena Cava and Physician Gestalt in the Emergency Department of an Urban Public Hospital in Sub-Saharan Africa
Source: PLoS One. 2016 Sep 27;11(9):e0162772. doi: 10.1371/journal.pone.0162772 (PMC5038941; doi:10.1371/journal.pone.0162772)
Supplement: S1 File — (PDF) [file pone.0162772.s001.pdf]

IVC_no	Vol_depletion	Main	Physical_exam	Age	Sex	Intubation	Cib	MAPb	Pulse_b	CI_500cc	MAP_500cc	Pulse_500cc	CI_1000cc	MAP_1000cc	Pulse_1000cc	CI_1500cc	MAP_1500cc	Pulse_1500cc	CI_2000	MAP_2000cc
1	mild	sepsis	Tachycardia	61	0	yes	30	74	110	20	75	110	30	77	100	4	78	92		
2	mild	hemorrhage	hypotension	26	1	no	12	74	111	6	75	108	21	78	102	14	80	96		
3	severe	hemorrhage	hypotension	28	0	no	55	58	102	46	69	101	26	73	98	21	79	96		
4	mild	poor intake	dryness of mucus membrane	36	1	no	50	69	92	44	83	90	9	87	86					
5	mild	vomiting	Tachycardia	56	1	no	46	80	99	40	81	98	24	85	96					
6	mild	hemorrhage	Tachycardia	26	0	no	43	81	109	9	84	106	21	89	92					
7	mild	hemorrhage	hypotension	36	0	no	16	65	104	12	65	100	27	67	101	25	80	98		
8	mild	diarrhoea	Tachycardia	43	1	no	42	81	102	30	86	98								
9	mild	diarrhoea	Tachycardia	33	0	no	35	87	114	29	87	108								
10	severe	hemorrhage	hypotension	42	1	no	61	56	108	45	64	106	31	72	96	26	67	92		
11	moderate	hemorrhage	hypotension	32	1	no	58	59	109	50	65	102	12	78	98	9	79	90		
12	mild	hemorrhage	dryness of mucus membrane	31	0	no	35	80	112	12	81	111								
13	mild	hemorrhage	dryness of mucus membrane	28	1	no	30	82	102	13	81	102								
14	mild	vomiting	dryness of mucus membrane	28	0	no	35	74	88	26	74	86								
15	mild	poor intake	dryness of mucus membrane	29	1	no	30	82	94	16	80	90								
16	mild	sepsis	Tachycardia	61	0	yes	28	74	110	21	75	110								
17	mild	poor intake	dryness of mucus membrane	19	0	no	26	72	88	25	80	86								
18	moderate	hemorrhage	hypotension	36	1	no	58	67	112	49	79	110	19	84	108	14	82	109		
19	mild	poor intake	dryness of mucus membrane	41	1	no	24	83	96	24	85	104	30	87	100	28	87	99		
19	mild	vomiting	Tachycardia	36	1	no	35	82	114	33	80	110	26	82	106					
20	severe	hemorrhage	Tachycardia	32	1	no	63	66	104	50	84	104	36	86	101	27	85	98		
21	mild	diarrhoea	dryness of mucus membrane	42	1	no	29	82	92	17	84	94								
22	mild	sepsis	Tachycardia	66	0	no	52	75	112	46	83	112	29	86	108	20	91	106	14	95
23	mild	hemorrhage	Tachycardia	26	1	no	26	80	110	20	81	109	22	86	100	24	85	96		
24	severe	hemorrhage	hypotension	34	0	no	60	59	114	45	58	110	30	72	108	24	75	100	17	80
25	severe	sepsis	hypotension	44	1	no	59	54	104	44	64	101	44	74	99	33	77	94	28	81
26	mild	poor intake	dryness of mucus membrane	36	0	no	30	87	88	23	84	86	25	85	86	14	87	79		
27	severe	hemorrhage	hypotension	26	0	no	84	53	104	47	61	101	37	69	100	26	72	99	14	74
28	mild	hemorrhage	Tachycardia	26	1	no	32	84	109	17	82	108	33	85	100					
29	mild	poor intake	dryness of mucus membrane	29	1	no	30	79	94	17	79	90	15	94	88					
30	severe	hemorrhage	hypotension	34	0	no	55	60	121	48	70	120	25	74	116	23	80	108		
31	mild	sepsis	hypotension	46	1	no	52	69	111	43	80	108	15	83	98					
32	moderate	vomiting	hypotension	50	0	no	56	55	141	47	71	131	20	68	124	15	72	116		
33	mild	poor intake	dryness of mucus membrane	26	0	no	27	79	88	27	81	86	20	83	84	8	85	80		
34	mild	hemorrhage	Tachycardia	34	1	no	31	70	108	32	72	98	40	75	90	12	75	88		
35	mild	hemorrhage	hypotension	43	1	no	55	79	94	49	81	92	13	84	89					
36	severe	hemorrhage	dryness of mucus membrane	26	1	no	73	61	104	40	73	98	26	76	92					
37	severe	hemorrhage	hypotension	49	0	no	72	63	89	50	68	82	27	69	80					
38	moderate	hemorrhage	hypotension	21	1	no	54	61	88	47	78	92	11	80	86					
39	mild	poor intake	dryness of mucus membrane	40	1	no	25	84	90	19	87	89								
40	mild	hemorrhage	dryness of mucus membrane	22	1	no	28	70	60	16	78	66								
41	severe	hemorrhage	hypotension	32	1	no	54	57	104	41	67	101	21	73	101	19	78	96	18	79
42	mild	poor intake	dryness of mucus membrane	49	0	no	49	73	88	29	82	86	22	87	76					
43	severe	hemorrhage	hypotension	21	0	no	46	63	96	40	77	96	31	81	92	27	83	82		
44	mild	vomiting	dryness of mucus membrane	26	1	no	25	84	82	25	85	86	41	85	86	21	87	84		
45	moderate	hemorrhage	hypotension	36	0	no	58	57	84	46	69	82	18	72	82	15	74	78		
46	mild	hemorrhage	Tachycardia	29	1	no	37	77	102	23	78	100	35	81	96	10	81	94		
47	severe	hemorrhage	hypotension	29	0	no	52	70	102	45	71	100	11	73	100	9	75	98	6	76
48	severe	hemorrhage	hypotension	39	1	no	55	67	98	46	88	98	20	93	96	14	91	94		
49	mild	hemorrhage	Tachycardia	26	0	no	49	62	102	13	61	102	14	66	100	10	72	98	8	79
50	mild	diarrhoea	Tachycardia	43	1	no	24	77	98	11	81	92	16	83	86					
51	mild	sepsis	Tachycardia	24	0	no	12	79	82	8	80	80								
52	mild	hemorrhage	hypotension	30	1	no	22	57	106	18	57	104								
53	severe	hemorrhage	Tachycardia	36	0	no	63	56	114	48	77	109	11	80	106	7	83	102		
54	mild	poor intake	Tachycardia	24	1	no	13	73	112	18	84	108	31	86	104					
55	moderate	hemorrhage	Tachycardia	26	0	no	46	64	114	34	78	111	17	81	110	7	83	104		
56	mild	poor intake	Tachycardia	21	1	no	31	74	105	26	79	102	25	80	100					
57	moderate	hemorrhage	dryness of mucus membrane	26	0	no	12	79	90	39	79	86	12	82	80					
58	severe	vomiting	hypotension	31	0	no	68	61	106	53	75	104	30	81	100					
59	mild	hemorrhage	Tachycardia	22	0	no	35	75	106	33	75	104	10	79	102					
60	mild	hemorrhage	dryness of mucus membrane	24	1	no	16	101	131	17	103	111								
60	severe	hemorrhage	hypotension	25	0	no	57	61	89	52	75	86	18	77	82					
61	mild	vomiting	dryness of mucus membrane	19	0	no	23	83	88	13	83	102								
62	mild	sepsis	dryness of mucus membrane	40	1	no	30	85	121	11	78	102								
63	mild	hemorrhage	dryness of mucus membrane	40	0	no	23	79	89	17	79	104								
64	mild	hemorrhage	dryness of mucus membrane	42	1	no	28	88	84	20	89	114								
65	moderate	hemorrhage	dryness of mucus membrane	33	1	no	24	67	106	19	67	106								
66	mild	hemorrhage	dryness of mucus membrane	34	1	no	26	85	98	22	87	100								
67	severe	sepsis	dryness of mucus membrane	25	0	no	39	84	96	24	84	112								
68	mild	hemorrhage	Tachycardia	19	0	no	27	77	114	18	79	100								
69	moderate	vomiting	Tachycardia	40	1	no	24	81	111	21	82	104								
70	mild	hemorrhage	dryness of mucus membrane	40	0	no	33	86	82	21	86	88								
71	mild	vomiting	dryness of mucus membrane	42	1	no	21	81	90	16	81	92								
74	severe	hemorrhage	hypotension	38	0	yes	54	61	106	46	78	102	32	79	99	22	83	92		
75	mild	sepsis	dryness of mucus membrane	21	0	no	34	85	86	25	87	24	40	93	72					
76	severe	hemorrhage	hypotension	65	0	no	58	60	92	52	73	90	12	76	80	9	78	90		
77	moderate	sepsis	hypotension	34	1	no	53	57	102	48	70	98	27	72	96					
78	severe	sepsis	hypotension	56	1	no	76	61	99	48	68	99	25	70	96	22	73	92		
79	severe	hemorrhage	Tachycardia	48	0	no	59	66	114	44	61	109	32	78	101					
80	mild	sepsis	hypotension	61	1	no	51	70	94	39	70	92	35	75	88					
81	severe	hemorrhage	hypotension	36	0	yes	53	59	114	46	74	111	36	78	99					
82	moderate	hemorrhage	Tachycardia	22	0	no	32	77	109	23	79	98	12	80	96	15	83	90		
83	mild	vomiting	hypotension	28	0	no	13	86	111	8	88	108								
84	mild	diarrhoea	dryness of mucus membrane	51	0	no	17	89	90	13	90	86								
85	mild	hemorrhage	dryness of mucus membrane	39	1	no	17	100	124	13	102	120								
86	mild	hemorrhage	dryness of mucus membrane	50	0	no	17	73	112	8	77	108								
87	mild	hemorrhage	dryness of mucus membrane	26	1	no	14	85	94	9	85	72								
88	mild	hemorrhage	hypotension	23	0	no	23	68	104	12	69	92								
90	mild	hemorrhage	Tachycardia	21	1	no	30	70	101	19	70	111								
91	mild	poor intake	hypotension	34	1	no	26	65	58	19	71	52	42	74	50					
92	moderate	sepsis	Tachycardia	40	1	no	56	59	102	50	67	98	24	74	96	19	79	96		
93	mild	poor intake	dryness of mucus membrane	67	0	no	27	84	76	23	84	72								
94	mild	hemorrhage	hypotension	25	1	no	51	79	60	45	79	54	12	80	52					
95	mild	poor intake	decreased skin turgor	19	0	no	22	95	89	17	97	89								
96	mild	hemorrhage	Tachycardia	19	0	no	48	73	102	12	77	96	30	79	96					
97	moderate	hemorrhage	Tachycardia	66	1	no	55	71	100	43	85	102	23	82	100					
98	mild	poor intake	dryness of mucus membrane	44	0	no	14	98	94	10	99	92								
99	severe	hemorrhage	hypotension	36	1	yes	54	54	108	46	55	102								
100	mild	poor intake	dryness of mucus membrane	41	1	no	21	81	88	13	85	86								
101	severe	diarrhoea	hypotension	19	1	no	54	67	104	62	75	102	25	76	102					
102	severe	vomiting	hypotension	23	0	no	79	56	99	48	62	92	37	65	92					
103	moderate	hemorrhage	Tachycardia	34	1	no	54	64	106	42	79	102	26	81	100					
104	mild	hemorrhage	Tachycardia	36	1	no	35	82	126	37	83	122	23	86	109	27	86	106		
105	severe	sepsis	hypotension	64	0	yes	67	57	54	60	72	50	17	71	54	13	77	59		
105	severe	sepsis	Tachycardia	64	0	yes	88	56	54	60	75	50	38	71	54	27	71	98	18	85
106	moderate	sepsis	Tachycardia	26	1	no	56	61	111	52	74	108	23	76	106					
107	mild	poor intake	Tachycardia	48	1	no	21	78	121	11	81	116	33	85	110					
108	mild	hemorrhage	Tachycardia	30	1	no	36	80	109	41	83	109	23	84	104					
109	mild	hemorrhage	Tachycardia	21	0	no	25	74	114	18	75	113	17	78	110	23	83	104		
110	severe	hemorrhage	Tachycardia	24	1	no	76	76	111	50	77	109	24	81	109					
111	mild	hemorrhage	Tachycardia	31	0	no	29	77	112	12	76	111								
112	severe	hemorrhage	hypotension	32	1	no	75	69	109	43	73	106	25	77	104					
113	severe	sepsis	hypotension	43	0	no	81	59	114	46	69	112	15	72	110					
114	mild	hemorrhage	dryness of mucus membrane	27	0	yes	27	77	96	21	81	94								
115	mild	hemorrhage	Tachycardia	28	1	no	29	75	102	13	76	102	16	80	98					
116	moderate	sepsis	Tachycardia	33	0	no	54	65	106	54	75	106	21	79	102					
117	mild	sepsis	hypotension	55	0	no	52	71	79	42	73	74	22	74	72					
118	severe	hemorrhage	hypotension	44	1	no	55	61	102	42	70	101	42	75	100	32	79	98		
119	mild	hemorrhage	hypotension	39	0	no	52	77	114	51	77	114	14	77	108	10	79	101		
120	mild	hemorrhage	dryness of mucus membrane	31	0	no	23	83	84	19	83	84	27	86	86					
121	mild	diarrhoea	dryness of mucus membrane	18	0	no	30	84	92	17	87	94								
122	severe	sepsis	hypotension	41	1	no	53	59	112	47	67	92	20	70	92					
123	mild	hemorrhage	Tachycardia	33	1	no	36	79	104	13	82	102								
124	severe	sepsis	hypotension	24	0	no	74	62	102	49	70	99	21	74	98					

125	mild	hemorrhage	Tachycardia	29	0	no	25	90	111	15	93	108								
126	severe	sepsis	hypotension	54	1	no	81	61	104	44	70	98	29	74	96					
127	mild	hemorrhage	Tachycardia	24	1	no	26	85	111	26	87	109	22	89	104					
128	mild	hemorrhage	dryness of mucus membrane	27	1	no	47	79	92	46	79	82	16	82	80					
129	moderate	poor intake	dryness of mucus membrane	42	1	no	53	73	96	50	91	94	16	84	92					
130	moderate	sepsis	Tachycardia	23	0	no	43	80	109	22	83	108	26	84	104					
131	moderate	hemorrhage	hypotension	51	0	no	55	77	98	45	77	94	14	78	94					
132	mild	hemorrhage	hypotension	36	0	no	52	72	82	47	75	72	8	79	68					
133	moderate	vomiting	hypotension	34	1	no	56	79	99	50	80	94	36	83	92					
134	moderate	poor intake	hypotension	66	0	no	58	74	112	42	74	112	22	82	109					
135	mild	hemorrhage	hypotension	44	0	no	52	68	116	45	73	114	27	75	111					
136	mild	hemorrhage	dryness of mucus membrane	23	1	no	36	75	68	20	79	62	23	80	68					
137	moderate	hemorrhage	hypotension	43	1	no	57	60	98	40	79	96	38	82	96					
138	severe	hemorrhage	hypotension	30	1	no	55	62	109	47	77	108	45	81	102					
139	mild	sepsis	Tachycardia	26	1	no	36	72	104	41	75	102	11	76	101					
140	severe	hemorrhage	hypotension	30	0	no	63	61	109	48	73	104	24	74	106	18	76	102		
141	severe	hemorrhage	Tachycardia	44	1	no	64	70	114	50	87	114	23	91	112					
142	severe	hemorrhage	hypotension	33	0	no	75	70	112	46	74	111	25	77	111					
143	mild	hemorrhage	Tachycardia	24	1	no	28	85	114	24	89	112								
144	severe	poor intake	hypotension	46	1	no	65	63	66	58	74	72	53	77	72					
145	mild	vomiting	dryness of mucus membrane	21	0	no	27	79	88	26	84	84	27	86	82					
146	severe	hemorrhage	dryness of mucus membrane	34	0	no	66	71	88	55	79	88	23	80	86					
147	mild	vomiting	decreased skin turgor	28	0	no	30	74	88	26	76	86	27	79	84					
148	mild	diarrhoea	decreased skin turgor	40	1	no	35	91	86	22	92	88								
149	severe	hemorrhage	hypotension	28	0	no	72	79	94	45	79	94	27	81	92					
150	mild	poor intake	Tachycardia	56	1	no	19	77	101	19	79	89								
151	moderate	hemorrhage	hypotension	44	1	no	59	80	116	44	81	109	37	83	101					
152	moderate	diarrhoea	Tachycardia	39	0	no	46	62	111	29	73	109	19	77	109					
153	severe	sepsis	hypotension	54	1	no	82	63	116	61	80	108	24	82	106					
154	mild	poor intake	Tachycardia	49	1	no	19	100	131	17	105	122								
155	severe	hemorrhage	hypotension	33	0	no	54	76	113	49	76	111	2	78	109					
156	severe	hemorrhage	hypotension	59	0	no	67	66	122	56	78	122	28	79	118					
157	moderate	poor intake	Tachycardia	26	0	no	40	81	119	15	82	119	31	87	108					
158	mild	hemorrhage	Tachycardia	58	1	no	35	99	109	17	103	108								
159	severe	sepsis	hypotension	45	1	no	77	62	116	50	80	104	31	89	108					
160	mild	poor intake	Tachycardia	64	0	no	30	81	114	24	87	112								
161	mild	poor intake	Tachycardia	38	0	no	38	76	111	25	79	109	21	80	109					
162	severe	poor intake	Tachycardia	44	0	no	87	56	88	55	79	88	23	80	86	18	76	102	12	89
163	severe	poor intake	Tachycardia	23	0	no	57	69	114	46	69	112	15	72	110	9	83	102	5	94
164	severe	poor intake	Tachycardia	34	0	no	60	68	102	51	77	100	34	78	96					
165	severe	hemorrhage	dryness of mucus membrane	53	1	no	84	60	102	40	83	102	29	85	100	24	88	98	18	81
166	severe	vomiting	decreased skin turgor	19	0	yes	63	56	99	48	74	92	37	74	92					
167	severe	hemorrhage	Tachycardia	45	0	no	65	63	112	46	74	111	22	77	111					
168	severe	poor intake	dryness of mucus membrane	46	1	no	72	65	66	58	74	72	54	77	72					
169	severe	hemorrhage	Tachycardia	32	1	no	83	62	104	40	73	98	9	76	92					
170	severe	hemorrhage	Tachycardia	44	1	no	54	70	114	50	88	114	23	92	112					
171	severe	hemorrhage	hypotension	45	1	yes	61	72	104	50	84	104	36	86	101	17	87	98	10	98
172	severe	hemorrhage	dryness of mucus membrane	30	0	yes	50	58	109	48	73	104	27	74	106					
173	severe	hemorrhage	Tachycardia	23	0	no	58	60	114	48	77	109	16	80	106					
174	severe	hemorrhage	hypotension	19	0	no	78	66	94	45	76	94	27	80	92	18	86	98	15	81
175	severe	hemorrhage	dryness of mucus membrane	32	1	no	60	63	109	43	73	106	25	77	104	21	96	98	19	92
176	severe	hemorrhage	hypotension	33	1	yes	61	66	108	45	64	106	31	72	96					
177	severe	sepsis	hypotension	44	1	yes	77	68	118	58	75	72	29	79	102					
178	severe	sepsis	hypotension	66	1	no	53	66	99	40	73	98	25	80	96					
179	severe	hemorrhage	hypotension	78	0	no	62	70	89	50	87	114	27	83	80	23	96	98	20	81
180	severe	hemorrhage	hypotension	34	0	no	85	65	114	50	84	104	77	73	108					
181	severe	hemorrhage	Tachycardia	46	1	no	42	78	111	38	81	104	24	93	109					
182	moderate	vomiting	hypotension	34	0	no	59	66	99	51	81	94	36	83	92					
183	severe	hemorrhage	hypotension	33	0	no	60	57	82	46	76	84	31	78	68					
184	severe	hemorrhage	dryness of mucus membrane	67	1	yes	59	78	116	47	79	109	23	80	104	19	83	100		
185	moderate	sepsis	Tachycardia	26	0	no	65	68	102	53	70	98	24	74	96	19	79	96		
186	severe	hemorrhage	Tachycardia	24	0	no	47	70	121	58	70	109	19	76	101					
187	moderate	sepsis	hypotension	34	0	no	48	80	100	56	81	108	23	83	106					
188	moderate	vomiting	hypotension	42	1	no	56	65	12	42	72	131	20	68	124	15	72	116		
189	severe	hemorrhage	hypotension	40	0	yes	56	65	89	47	87	98	17	93	96	14	90	94		
190	severe	hemorrhage	hypotension	26	0	yes	59	58	89	47	78	94	14	78	94					
191	severe	hemorrhage	dryness of mucus membrane	29	0	no	50	72	108	38	73	101	26	75	98	21	77	96		
192	moderate	hemorrhage	hypotension	21	1	no	56	68	109	42	79	108	45	81	102					
193	severe	hemorrhage	hypotension	41	1	no	58	60	87	46	84	92	17	84	89					
194	severe	hemorrhage	decreased skin turgor	43	1	yes	45	56	98	45	71	101	39	75	100	33	79	98		
195	moderate	hemorrhage	Tachycardia	28	1	yes	57	63	100	45	87	102	18	92	100					
196	severe	hemorrhage	Tachycardia	31	1	no	53	58	120	49	70	120	33	74	116	25	82	108		
197	moderate	diarrhoea	Tachycardia	19	0	no	57	58	110	45	80	106	22	79	104					
198	moderate	hemorrhage	Tachycardia	48	1	no	54	62	78	44	78	92	11	80	86					
199	severe	poor intake	dryness of mucus membrane	41	0	no	54	67	96	47	88	88	42	93	86					
200	severe	hemorrhage	hypotension	32	1	no	57	63	102	49	70	101	27	73	101	18	78	96	13	79
201	severe	hemorrhage	hypotension	34	1	no	57	74	102	47	82	101	26	81	96	18	82	96		
202	mild	vomiting	decreased skin turgor	26	0	no	46	81	98	38	88	104	12	89	102					
203	severe	poor intake	hypotension	34	0	no	66	60	102	45	77	100	34	78	96					
204	severe	vomiting	hypotension	28	1	no	67	64	102	39	83	102	27	85	100					
205	severe	hemorrhage	hypotension	28	0	no	66	59	99	43	74	92	36	76	92					
206	severe	hemorrhage	hypotension	31	1	no	63	69	112	45	74	111	27	77	111					
207	moderate	hemorrhage	hypotension	42	0	yes	54	61	66	59	74	72	53	77	92					
208	moderate	hemorrhage	dryness of mucus membrane	24	1	yes	56	62	104	44	73	98	27	83	92					
209	severe	hemorrhage	Tachycardia	56	1	no	70	63	114	52	86	114	18	91	112					
210	severe	vomiting	Tachycardia	67	0	no	61	66	104	46	85	104	35	86	101					
211	severe	poor intake	hypotension	89	0	no	65	62	109	49	79	104	23	79	106					
212	severe	vomiting	decreased skin turgor	61	0	no	64	60	114	48	80	109	9	81	86					
213	severe	poor intake	Tachycardia	54	0	no	75	63	94	35	78	94	27	81	88					
214	moderate	hemorrhage	Tachycardia	19	1	no	74	65	109	40	75	106	27	77	101					
215	severe	hemorrhage	Tachycardia	24	1	no	62	59	108	53	68	106	34	77	96					
216	severe	poor intake	Tachycardia	23	1	yes	79	67	118	42	76	106	29	81	100					
217	moderate	hemorrhage	dryness of mucus membrane	66	1	no	82	58	99	49	78	99	25	81	96					
218	severe	hemorrhage	hypotension	49	0	no	59	64	89	54	79	82	27	84	100					
219	severe	hemorrhage	hypotension	34	0	no	58	57	114	42	59	110	30	75	92					
220	severe	hemorrhage	Tachycardia	24	1	no	70	69	111	45	77	109	24	93	100					
221	severe	hemorrhage	hypotension	33	1	no	56	57	82	46	77	84	31	78	68					
222	severe	hemorrhage	hypotension	51	1	no	54	59	107	42	65	101	34	71	99	28	78	92	24	81
223	moderate	poor intake	dryness of mucus membrane	52	0	no	54	68	92	44	85	88	42	93	86					
224	severe	sepsis	hypotension	62	0	no	58	58	122	39	71	110	38	77	108	19	81	104	15	82
225	severe	hemorrhage	hypotension	34	0	no	76	62	102	51	77	100	34	78	96					
226	severe	hemorrhage	hypotension	34	1	no	54	67	108	43	78	101	21	81	96	19	82	96		
227	mild	hemorrhage	hypotension	19	0	no	48	71	112	27	72	104	21	74	100	16	78	100		
228	mild	hemorrhage	hypotension	32	1	no	11	89	106	14	91	104	10	92	104	10	95	100	9	97
229	severe	sepsis	hypotension	41	1	yes	79	76	118	42	77	106	29	79	102	22	82	102	16	86
230	mild	diarrhoea	decreased skin turgor	24	0	no	9	90	82	23	93	80	23	98	76					
231	mild	hemorrhage	Tachycardia	71	0	no	32	89	108	14	90	106	13	91	106	12	92	100		
232	mild	vomiting	dryness of mucus membrane	61	1	no	27	85	90	25	89	90								
233	mild	sepsis	hypotension	34	1	no	13	84	124	17	85	120	26	88	118	22	90	114	18	92
234	severe	hemorrhage	hypotension	32	1	no	69	57	121	54	69	120	22	77	114	13	82	110	11	83
235	moderate	diarrhoea	Tachycardia	34	1	no	55	68	112	43	79	106	22	79	104					
236	mild	hemorrhage	Tachycardia	42	0	no	40	93	102	38	96	96	15	98	96					
237	severe	hemorrhage	hypotension	19	0	no	56	50	116	49	73	109	23	77	104	19	82	100		
238	severe	hemorrhage	hypotension	23	0	no	56	49	121	57	55	109	19	58	101					
239	mild	vomiting	decreased skin turgor	26	0	no	54	74	106	44	87	104	13	89	102					
240	mild	hemorrhage	Tachycardia	42	1	no	25	82	104	34	87	20	11	92	22	8	95	96		
241	severe	hemorrhage	hypotension	53	1	no	65	81	102	40	83	102	29	85	100	21	90	98		
242	mild	vomiting	Tachycardia	28	0	no	23	81	106	11	73	104	26	82	102					
242	mild	hemorrhage	Tachycardia	33	1	no	38	82	104	35	82	102								
243	mild	hemorrhage	hypotension	34	1	no	28	80	58	24	68	52								
243	moderate	hemorrhage	Tachycardia	44	1	no	47	80	104	17	80	108	26	89	100					
244	mild	hemorrhage	Tachycardia	25	0	no	34	88	111	29	90	109								
245	mild	diarrhoea	Tachycardia	43	1	no	31	87	110	23	89	109								
246	mild	poor intake	dryness of mucus membrane	32	1	no	25	86	90	23	88	90								
247	moderate	poor intake	dryness of mucus membrane	26	1	no	30	84	76	13	85	72								

248	mild	poor intake	dryness of mucus membrane	40	1	no	30	81	88	27	83	86								
249	mild	vomiting	dryness of mucus membrane	42	1	no	26	79	88	26	96	84								
250	mild	hemorrhage	dryness of mucus membrane	34	1	yes	33	78	96	21	78	94								
251	mild	hemorrhage	Tachycardia	24	1	no	32	89	114	24	91	112								
252	mild	hemorrhage	dryness of mucus membrane	26	1	no	32	90	60	16	89	66								
253	mild	vomiting	Tachycardia	22	1	no	30	84	108	25	88	92								
254	mild	sepsis	Tachycardia	61	1	no	48	77	106	22	84	100								
255	moderate	vomiting	hypotension	33	1	no	52	83	112	47	79	106	33	91	108					
256	mild	hemorrhage	Tachycardia	34	1	no	80	61	100	59	77	98	26	81	98					
257	mild	hemorrhage	Tachycardia	36	0	no	34	82	111	26	80	101	18	83	104					
259	mild	vomiting	Tachycardia	40	1	no	29	77	108	20	78	92								
261	mild	poor intake	Tachycardia	40	0	no	31	81	114	27	82	112								
262	severe	hemorrhage	hypotension	26	1	no	81	58	114	48	69	114	15	72	112	11	77	108	10	79
263	moderate	hemorrhage	hypotension	27	0	no	51	70	82	21	71	96	24	73	78					
264	moderate	diarrhoea	dryness of mucus membrane	30	1	no	75	84	78	39	92	78	39	91	77	32	96	82		
265	severe	hemorrhage	hypotension	50	1	no	76	63	151	60	71	169	49	75	166	47	72	164	21	79
266	severe	hemorrhage	hypotension	26	1	no	83	73	112	56	79	116	63	79	94	68	82	106	50	88
267	moderate	vomiting	dryness of mucus membrane	34	0	no	58	82	99	47	87	88	44	93	86					
268	mild	sepsis	hypotension	41	0	no	65	74	111	17	80	108	24	83	98					
269	severe	hemorrhage	hypotension	28	1	no	63	71	134	34	75	132	31	80	132					
270	moderate	sepsis	hypotension	29	1	yes	71	81	96	33	85	90	29	97	72					
271	severe	hemorrhage	hypotension	36	0	no	89	63	144	42	75	140	63	77	132	51	82	122	49	81
272	mild	hemorrhage	tachycardia	24	1	no	37	83	132	30	86	130	35	91	131					
273	mild	hemorrhage	hypotension	38	0	no	48	58	142	46	59	144								
274	mild	hemorrhage	tachycardia	28	0	no	67	96	134	11	106	132								
275	severe	hemorrhage	hypotension	38	1	no	35	71	125	23	71	130	32	75	128					
276	mild	sepsis	dryness of mucus membrane	66	0	no	75	74	88	66	79	86	72	85	79					
277	moderate	hemorrhage	tachycardia	32	1	no	83	78	98	44	79	96	55	85	90					
278	severe	hemorrhage	hypotension	44	1	yes	51	61	108	34	64	101	36	74	99					
279	moderate	hemorrhage	hypotension	29	0	no	30	79	94	31	82	92	24	83	88					
280	mild	vomiting	tachycardia	48	0	no	37	85	99	17	94	92								
281	severe	hemorrhage	hypotension	29	0	yes	68	71	112	50	73	108	45	80	119					
282	mild	sepsis	dryness of mucus membrane	32	1	no	41	82	72	37	83	68								
283	mild	vomiting	tachycardia	48	1	no	37	87	92	27	88	90								
284	mild	hemorrhage	hypotension	66	0	no	55	84	82	28	85	71	30	92	68					
285	severe	hemorrhage	hypotension	29	0	yes	32	61	108	50	66	101	11	76	62					
286	severe	hemorrhage	hypotension	32	0	yes	94	61	101	39	77	96	101	88	96	38	82	92		
287	mild	diarrhoea	dryness of mucus membrane	30	1	no	26	84	72	7	89	78								
288	moderate	vomiting	decreased skin turgor	27	1	no	6	81	76	36	81	80	10	87	76					
289	severe	hemorrhage	hypotension	23	1	yes	46	70	60	31	67	50								
290	moderate	sepsis	dryness of mucus membrane	33	1	no	33	87	82	38	87	80	26	93	72					
291	mild	sepsis	dryness of mucus membrane	49	1	yes	16	87	76	31	89	77	23	90	81					
292	mild	sepsis	dryness of mucus membrane	41	0	no	51	87	76	38	92	70								
293	severe	vomiting	dryness of mucus membrane	40	1	no	78	65	99	75	75	70								
294	mild	sepsis	decreased skin turgor	26	1	no	26	78	70	43	78	72	41	79	68					
295	mild	diarrhoea	dryness of mucus membrane	22	0	no	45	84	66	38	89	110	20	89	68					
296	mild	vomiting	dryness of mucus membrane	36	0	no	26	84	66	16	87	98								
297	moderate	hemorrhage	hypotension	23	1	no	42	76	92	40	77	90								
298	moderate	hemorrhage	hypotension	28	0	no	65	73	78	35	78	76	42	82	78					
299	moderate	sepsis	hypotension	21	0	no	46	77	82	16	78	81	14	77	82					
300	severe	hemorrhage	hypotension	19	0	no	60	76	88	59	80	62	35	82	66					
301	severe	hemorrhage	hypotension	24	1	no	66	53	54	61	58	46								
302	severe	hemorrhage	hypotension	26	0	yes	32	69	89	23	73	92								
303	moderate	sepsis	hypotension	49	0	yes	53	76	104	12	78	104								
304	severe	hemorrhage	hypotension	21	0	yes	42	71	128	24	75	126	29	73	108	27	41	106	29	83
305	moderate	vomiting	hypotension	36	1	no	45	86		38	88		21	90		22	94			
306	moderate	vomiting	dryness of mucus membrane	41	0	no	28	79	106	29	79	104	17	84	106	15	85	92		
307	severe	sepsis	hypotension	52	0	no	36	70	140	20	75	106	18	82	92					
308	severe	sepsis	dryness of mucus membrane	54	1	no	76	74	102	3	83	100	57	86	102	57	92	102		
309	moderate	hemorrhage	hypotension	34	0	no	32	73	102	16	85	90	11	90	92					
310	mild	hemorrhage	tachycardia	33	0	no	34	85	128	34	85	102								
311	moderate	hemorrhage	tachycardia	29	0	no	48	77	104	14	80	102	52	78	102					
312	mild	vomiting	dryness of mucus membrane	35	0	no	31	85	92	22	91	90								
313	mild	vomiting	tachycardia	46	0	no	33	88	102	17	94	66								
314	moderate	hemorrhage	decreased skin turgor	30	1	no	55	85	82	58	85	71	30	94	68	64	91	66		
315	moderate	hemorrhage	tachycardia	34	1	no	69	84	121	23	85	90								
316	moderate	hemorrhage	tachycardia	21	0	no	31	78	106	27	78	102	14	80	100	17	79	96		
317	mild	diarrhoea	dryness of mucus membrane	54	1		32	78	99	20	84	92								
318	moderate	hemorrhage	hypotension	22	0	no	69	74	102	50	77	102	42	81	98	25	83	96		
319	mild	diarrhoea	dryness of mucus membrane	38	1	no	50	78	92	15	74	90	49	79	96					
320	mild	vomiting	tachycardia	26	1	no	27	77	126	17	79	102								
321	moderate	hemorrhage	hypotension	36	1	no	33	74	102	27	75	104	35	81	106	39	83	98		
322	moderate	vomiting	hypotension	44	1	no	46	60	90	6	71	66	27	78	50					
323	severe	hemorrhage	hypotension	20	0	no	30	53	66	35	56	60								
324	severe	hemorrhage	hypotension	29	1	yes	71	63	50	31	67	40								
325	moderate	sepsis	dryness of mucus membrane	56	1	no	40	81	90	23	82	98	27	84	84	25	92	80		
326	moderate	sepsis	hypotension	44	1	no	54	67	109	13	70	111	26	73	109	32	77	94		
327	mild	vomiting	dryness of mucus membrane	29	1	no	38	82	66	18	80	72	11	87	71					
328	severe	vomiting	hypotension	32	1	no	76	75		31	66		17	66						
329	severe	hemorrhage	hypotension	32	1	no	43	59	52	17	53	44								
330	moderate	sepsis	dryness of mucus membrane	52	1	no	6	81	82	31	79	80	32	81	80	16	84	82		
331	moderate	vomiting	dryness of mucus membrane	42	1	no	44	87	92	20	85	89	17	91	82					
332	mild	hemorrhage	dryness of mucus membrane	21	1	no	26	87	78	50	95	66								
333	mild	vomiting	dryness of mucus membrane	32	0	no	17	97	72	18	101	84								
334	moderate	sepsis	hypotension	32	0	no	26	75	103	43	77	96	20	77	92					
335	moderate	sepsis	dryness of mucus membrane	24	1	no	36	82	102	19	83	98	22	83	98					
336	moderate	vomiting	tachycardia	36	0	no	43	79	104	38	81	104	6	83	102					
337	severe	hemorrhage	hypotension	44	1	no	74	66	144	52	77	146	60	77	109	52	82	111	49	88
338	moderate	sepsis	hypotension	61	0	no	51	108	108	8	119	111								
339	moderate	sepsis	hypotension	32	0	no	52	74	98	40	74	88	23	79	86					
340	mild	vomiting	tachycardia	36	0	no	23	78	90	30	80	92								
341	mild	sepsis	tachycardia	32	1	no	72	88	90	4	94	84								
342	severe	sepsis	hypotension	48	1	yes	22	71		7	73									
343	moderate	vomiting	dryness of mucus membrane	41	0	no	42	87	90	10	93	92	7	94	86					
344	moderate	hemorrhage	decreased skin turgor	36	0	no	31	75	84	14	77	82	19	83	82					
345	severe	hemorrhage	hypotension	40	1	no	95	62	144	46	72	140	54	74	140	50	81	132	49	82
346	severe	hemorrhage	hypotension	34	0	no	85	58	146	69	68	144	46	63	144	41	72	138	56	70
347	moderate	sepsis	dryness of mucus membrane	26	0	yes	28	75	101	19	78	86	39	80	84					
348	moderate	hemorrhage	hypotension	29	0	no	50	70	82	23	70	96	12	77	92					
349	moderate	vomiting	dryness of mucus membrane	34	1	no	75	85	74	50	87	76	43	104	72	41	92	82		
350	moderate	hemorrhage	hypotension	38	1	no	79	76	121	60	77	116	62	79	106	58	82	94		
351	moderate	hemorrhage	tachycardia	22	0	no	35	83	130	30	86	130	34	91	122					
352	severe	hemorrhage	hypotension	36	1	yes	84	63	141	59	74	136	54	78	138	51	82	128	52	85
353	severe	hemorrhage	hypotension	29	1	yes	77	67	132	63	70	130	59	77	130	47	81	121	48	81
354	moderate	vomiting	tachycardia	36	0	no	55	73	144	35	78	136	31	82	138	11	82	122		
355	moderate	hemorrhage	tachycardia	38	1	no	62	73	126	35	74	122	12	80	109					
356	severe	sepsis	hypotension	61	0	no	74	62	152	62	72	144	56	75	146	47	77	136	21	79
357	moderate	sepsis	tachycardia	32	0	no	54	78	101	14	80	100	16	82	96					
358	mild	sepsis	tachycardia	29	0	no	55	75	102	23	75	102	17	79	98					
359	mild	vomiting	dryness of mucus membrane	41	1	no	47	81	99	39	82	102	8	88	92					
360	moderate	hemorrhage	hypotension	38	0	no	15	76	92	17	77	92	20	79	88					
361	moderate	vomiting	dryness of mucus membrane	29	0	yes	47	74	92	8	81	90	23	82	88					
362	moderate	hemorrhage	hypotension	38	0	no	20	70	108	40	108	108	18	76	106	8	78	100		
363	moderate	sepsis	dryness of mucus membrane	62	1	no	25	80		20	82		14	83						
364	moderate	sepsis	dryness of mucus membrane	42	1	no	54	75	92	9	77	96	17	81	96					
